# Clinical profiles and antimicrobial resistance patterns of invasive *Salmonella* infections in children in China

**DOI:** 10.1007/s10096-022-04476-7

**Published:** 2022-08-30

**Authors:** Wen Song, Qingwen Shan, Yue Qiu, Xianyao Lin, Chunhui Zhu, Zhiqiang Zhuo, Caihong Wang, Jianning Tong, Rui Li, Chaomin Wan, Yu Zhu, Minxia Chen, Yi Xu, Daojiong Lin, Shouye Wu, Chunmei Jia, Huiling Gao, Junwen Yang, Shiyong Zhao, Mei Zeng, Collaborative Working Group of the Pediatric Subgroup of the China Society of Infectious Diseases

**Affiliations:** 1 Department of Infectious Diseases, Hangzhou Children’s Hospital, 195 Wenhui Road, Hangzhou, 310014 China; 2 Department of Hospital Infection Management, Affiliated Hangzhou First People’s Hospital, Zhejiang University School of Medicine, 261 Huansha Road, Hangzhou, 310006 China; 3 Department of Pediatrics, The First Affiliated Hospital of Guangxi Medical University, Nanning, China; 4 Department of Infectious Diseases, Children’s Hospital of Fudan University, 399 Wanyuan Road, Shanghai, 201102 China; 5 Department of Infectious Diseases, Jiangxi Provincial Children’s Hospital, Nanchang, China; 6 Department of Infectious Diseases, Xiamen Children’s Hospital, Xiamen, China; 7 Department of Pediatric, Gastroenterology and Infectious Diseases, Qingdao Women and Children’s Hospital, Qingdao, China; 8 Department of Pediatrics, Sichuan University West China Second Hospital (West China Women’s and Children’s Hospital), Chengdu, China; 9 Department of Infectious Diseases, Guangzhou Women and Children’s Medical Center, Guangzhou, China; 10 Department of Infectious Diseases, Hainan Women and Children’s Medical Center, Haikou, China; 11 Department of Pharmacy, The Forth Hospital of Baotou, Baotou, China; 12 Department of Microbiology Laboratory, Children’s Hospital Affiliated to Zhengzhou University (Henan Children’s Hospital), Zhengzhou, China

**Keywords:** Invasive nontyphoidal *Salmonella*, Typhoid fever, Children, China

## Abstract

Invasive *Salmonella* infections result in a significant burden of disease including morbidity, mortality, and financial cost in many countries. Besides typhoid fever, the clinical impact of non-typhoid *Salmonella* infections is increasingly recognized with the improvement of laboratory detection capacity and techniques. A retrospective multicenter study was conducted to analyze the clinical profiles and antimicrobial resistance patterns of invasive *Salmonella* infections in hospitalized children in China during 2016–2018. A total of 130 children with invasive *Salmonella* infections were included with the median age of 12 months (range: 1–144 months). Seventy-nine percent of cases occurred between May and October. Pneumonia was the most common comorbidity in 33 (25.4%) patients. Meningitis and septic arthritis caused by nontyphoidal *Salmonella* (NTS) infections occurred in 12 (9.2%) patients and 5 (3.8%) patients. Patients < 12 months (OR: 16.04) and with septic shock (OR: 23.4), vomit (OR: 13.33), convulsion (OR: 15.86), C-reactive protein (CRP) ≥ 40 g/L (OR: 5.56), and a higher level of procalcitonin (PCT) (OR: 1.05) on admission were statistically associated to an increased risk of developing meningitis. Compared to 114 patients with NTS infections*,* 16 patients with typhoid fever presented with higher levels of CRP and PCT (*P* < 0.05). The rates of resistance to ampicillin, sulfamethoxazole/trimethoprim, ciprofloxacin, and ceftriaxone among *Salmonella* Typhi and NTS isolates were 50% vs 57.3%, 9.1% vs 24.8%, 0% vs 11.2%, and 0% vs 9.9%, respectively. NTS has been the major cause of invasive *Salmonella* infections in Chinese children and can result in severe diseases. Antimicrobial resistance among NTS was more common.

## Introduction

Typhoid fever and other invasive salmonellosis continue to cause an estimated 14.8 million cases and > 200,000 deaths annually, largely affecting children in low- and middle-income countries [1]. Enteric fever, the collective term for typhoid and paratyphoid fevers, describes a systemic infection caused by *Salmonella enterica* serovar Typhi or Paratyphi A, B, or C [2]. Nontyphoidal *Salmonella* (NTS) serovars that typically cause self-limiting diarrhea can also cause invasive systemic infections [3]. Nowadays, invasive NTS infections, which cause an estimated 535,000 cases and > 77,000 deaths annually, are increasingly becoming an important public health threat in low- and middle-income countries, and have gained greater recognition as an important disease in children younger than 5 years [1, 4]. A global systematic review and meta-analysis showed that the pooled case-fatality ratio (CFR) was 17.1% in Africa, 14.0% in Asia, 9.9% in Europe, and 9.6% in the Americas [5]. In some Asian countries such as India [6], Thailand [7], and Vietnam [8], enteric fever remains a public health issue. In China, the average incidence of typhoid and paratyphoid was 1.03/100 000 during 2009–2013 and decreased markedly over year [9]. However, foodborne NTS gastroenteritis and outbreaks increasingly gained public attention and an estimated 70–80% of bacterial food poisoning is caused by *Salmonella* [10, 11]. A recent study focused on the molecular epidemiology and antimicrobial resistance of invasive *Salmonella* strains in China [12]. However, few studies have been designed to comprehensively describe the case series of invasive NTS disease from different regions of China. Therefore, we conducted a retrospective multicenter study to recognize the detailed clinical profiles of invasive *Salmonella* infections in Chinese children.

## Methods

### Participating hospitals and sites

Ten tertiary hospitals from different provinces in China participated in this study during 2016–2018: four hospitals are located in the Southeast or East of China (Hangzhou Children’s Hospital, Jiangxi Provincial Children’s Hospital, Xiamen Children’s Hospital, and Qingdao Women and Children’s Hospital), four hospitals are located in the Southwest or South region (West China Women’s and Children’s Hospital, The First Affiliated Hospital of Guangxi Medical University, Guangzhou Women and Children’s Medical Center, and Hainan Women and Children’s Medical Center), one hospital is located in the north region (The Forth Hospital of Baotou), and the other one hospital is located in the central China (Henan Children’s Hospital).

### Case definition

A case of invasive *Salmonella* infections is defined as a patient with symptoms consistent with an invasive bacterial infection, from whom culturing of a blood and/or other sterile body fluid (such as cerebrospinal fluid, pleural fluid, pericardial fluid, joint fluid, bone aspirate, or a deep tissue abscess) samples grew *Salmonella* Typhi, or *Salmonella* Paratyphi A, B, and C, or NTS serovar [13]. A case of meningitis is defined as a patient with clinical symptoms with leukocytosis of cerebrospinal fluid and a positive culture of cerebrospinal fluid culture and/or blood. A case of septic arthritis is defined as a patient with clinical symptoms such as swelling, redness, heat, and pain in a single joint associated with a decreased ability to move the joint and a positive culture of blood and/or synovial fluid.

### Data collection and definition of disease outcomes

Hospitalized pediatric patients < 18 years of age who had invasive *Salmonella* disease infections and whose medical records were available for complete review were included in this study, based on microbiology registries in departments of laboratory medicine. Two designated clinical investigators at each participating hospital reviewed the medical records case-by-case. A standard case report form was used to record sites of bacteria isolation, patient’s demographics, clinical symptoms, laboratory findings, and disease outcomes. The final outcomes were evaluated based on the discharge records and were classified as follows: (1) recovery or improvement, (2) death or hopeless discharge, (3) transfer to another hospital under either parents’ request or doctors’ counsel (but not expected to die). Outcome 2 was considered to be fatal. Cases classified as “transfer to another hospital” were considered missing data because the final outcomes could not be determined.

### Isolate identification and antimicrobial susceptibility testing

The clinical samples were processed following standard blood culture procedures. Species identification and antimicrobial susceptibility testing were performed at local hospital laboratories by automated systems such as Vitek or Phoenix, according to the Clinical and Laboratory Standards Institute (CLSI) performance guideline. Antimicrobial susceptibility testing was performed using minimum inhibitory concentrations (MICs) or the Kirby-Bauer disk diffusion method. The original results were interpreted according to the breakpoints of the CLSI standards [14]. The isolates were classified as resistant, intermediate, and sensitive. Isolates displaying intermediate susceptibility were not categorized as resistant in this study. Isolates resistant to three or more classes of antimicrobial agents were defined as multidrug resistant (MDR) [15].

### Statistical analysis

Age groups were stratified as < 12 months of age and ≥ 12 months of age. Statistical analysis was performed using the SPSS Software (version 23.0 for windows; SPSS Inc., Chicago, IL, USA). For continuous variables, non-normally distributed variables were presented as medians and interquartile ranges (IQRs) and were compared by means of the Mann–Whitney non-parametric test. Categorical variables were expressed as percentages and compared by means of Fisher’s exact test or the chi-square test, as appropriate. The univariate analysis to compare between the meningitis group and non-meningitis group was performed by binary logistic regression and the odds ratios (ORs) with 95% confidence intervals (CIs) were reported. Statistical significance was accepted at *P* ≤ 0.05.

## Results

### The demographic and clinical features of the children with invasive *Salmonella* infections

**Fig. 1:**
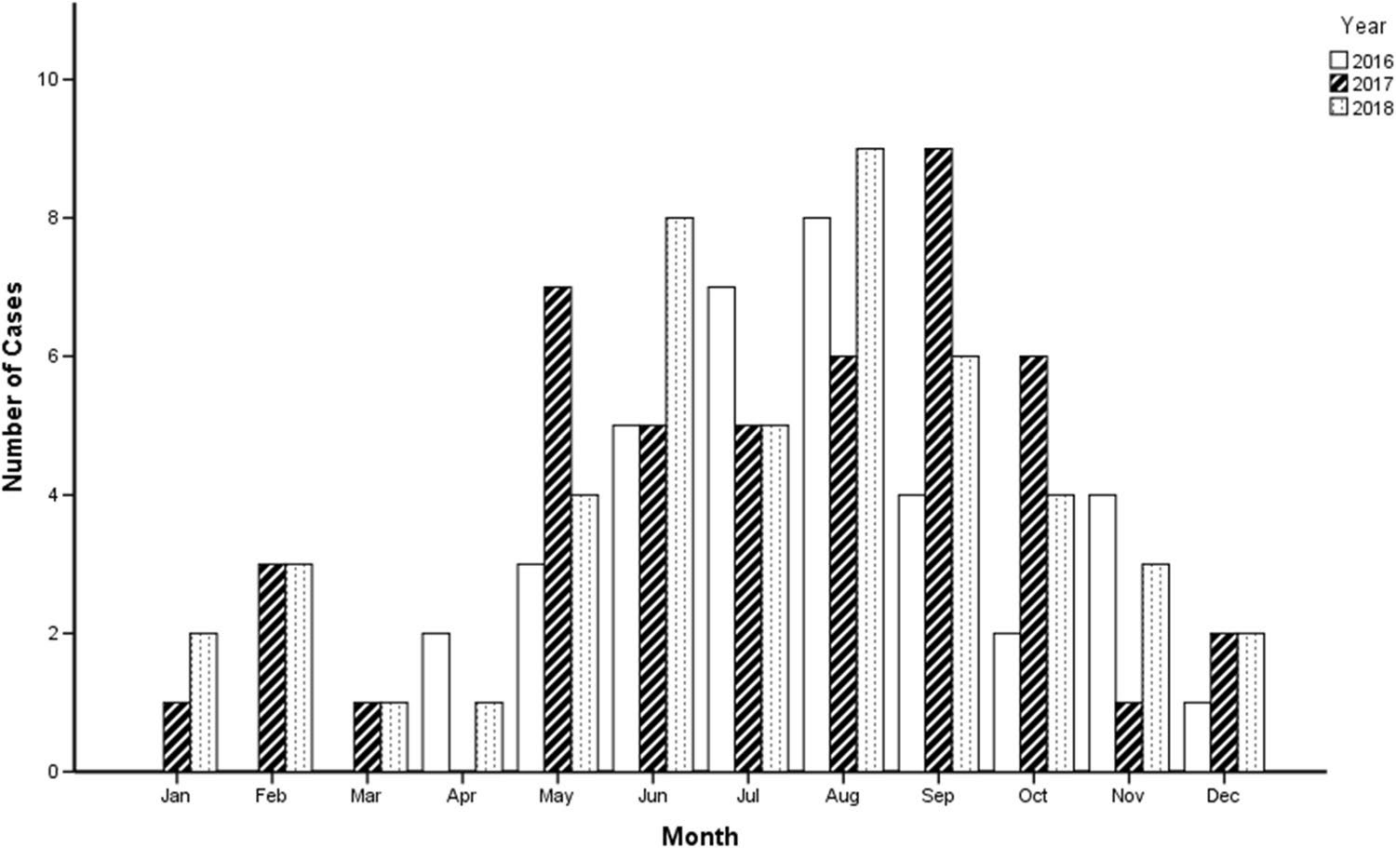
Seasonality of invasive *Salmonella* infections among inpatients by year

In total, 130 children with invasive *Salmonella* infections were included in the study. Of these patients, 119 (91.5%) had cultures recovered from blood alone, 5 (3.8%) from cerebrospinal fluid alone, 4 (3.1%) from both blood and cerebrospinal fluid, 1 (0.8%) from joint fluid, and 1 (0.8%) from bone aspirate. The most cases were from Jiangxi Provincial Children’s Hospital (44), followed by Henan Children’s Hospital (35), West China Women’s and Children’s Hospital (14), Xiamen Children’s Hospital (9), Hainan Women and Children’s Medical Center (9), The First Affiliated Hospital of Guangxi Medical University (7), Guangzhou Women and Children’s Medical Center (5), Hangzhou Children’s Hospital (4), Qingdao Women and Children’s Hospital (2), and The Forth Hospital of Baotou (1). Seventy-seven (59.2%) patients were males. Median age was 12 (IQR: 8–20.25, range: 1–144) months; 85.4% were younger than 36 months old. Seventy-nine percent of cases occurred between May and October (see Fig. 1).

Among the 130 patients, the three most common clinical symptoms were fever with body temperature ≥ 38℃ (118/130, 90.8%), diarrhea (43/130, 33.1%), and cough (37/130, 28.5%); 80.5% of febrile patients had maximum body temperature ≥ 39℃ for 5 days of median duration (IQR 2–7 days, range: 1–90 days) prior to hospital admission.

Twelve (9.2%) patients (median age: 4 months) had meningitis and 5 (3.8%) patients (median age: 19 months) had septic arthritis, for whom NTS serovars (5/5) were the causative pathogens. Six patients with meningitis had comorbidity of pneumonia (4/6), cardiac disease (1/6), and hematological malignancies (2/6). Patients with arthritis had no comorbidity. As shown in Table 1, we found that the patients with meningitis were younger (*P* < 0.001) and had longer length of hospital stay (*P* = 0.025) than those without meningitis. In addition, according to the clinical symptoms, septic shock (*P* = 0.022), vomit (*P* = 0.001), and convulsion (*P* < 0.001) were significantly associated with meningitis caused by *Salmonella* infection. Also, the analysis of laboratory findings showed that the levels of C-reactive protein (CRP) ≥ 40 g/L (*P* = 0.012) and evaluated procalcitonin (PCT) (*P* < 0.001) were more common among the patients with meningitis. According to the univariate analysis, the risk factors of meningitis were patients < 12 months of age (OR: 16.04, *P* = 0.009), septic shock (OR: 23.4, *P* = 0.013), vomit (OR: 13.33, *P* < 0.001), convulsion (OR: 15.86, *P* < 0.001), CRP ≥ 40 g/L (OR: 5.56, *P* = 0.009), and higher PCT (OR: 1.05, *P* = 0.013) (see Table 2).

**Table 1:** The clinical characteristics of patients with invasive *Salmonella* infections before administrated associated with meningitis

Parameters	Total	Meningitis group	Non-meningitis group	*P* value
	(*n* = 130)	(*n* = 12)	(*n* = 118)	
*Demographics*				
Age (month), median (IQR)	12 (8–20.25)	4 (1–8)	12 (9–21)	< 0.001*
Gender, male (%)	77 (59.2)	7 (58.3)	70 (59.3)	1.000
Comorbidities (%)	55 (42.3)	6 (50.0)	49 (41.5)	0.571
Length (days) of hospital stay, median (IQR)	10 (7–15.25)	28.5 (8.75–30.75)	10 (6.75–14)	0.025*
*Symptoms on admission*				
Fever (%)	118 (90.8)	12 (100)	106 (89.8)	0.602
Fever duration (day), median (IQR)	5 (2–7)	3 (1.25–5.75)	5 (2–7)	0.116
Septic shock (%)	3 (2.3)	2 (16.7)	1 (0.8)	0.022*
Rash (%)	15 (11.5)	0 (0)	15 (12.7)	0.358
Cough (%)	37 (28.5)	1 (8.3)	36 (30.5)	0.177
Respiratory distress (%)	5 (3.8)	1 (8.3)	4 (3.4)	0.389
Tachypnea (%)	8 (6.2)	0 (0)	8 (6.8)	1.000
Cyanosis (%)	2 (1.5)	1 (8.3)	1 (0.8)	0.177
Vomit (%)	11 (8.5)	5 (41.7)	6 (5.1)	0.001*
Diarrhea (%)	43 (33.1)	1 (8.3)	42 (35.6)	0.103
Convulsion (%)	13 (10)	6 (50)	7 (5.9)	< 0.001*
*Laboratory indicators on admission*				
Leukocyte < 4000 or > 15,000 count/mm^3^ (%)	43(35) ^a^	6(50)	37(33.3) ^a^	0.340
Neutropenia (%)	8 (6.5) ^a^	0 (0)	8 (7.2) ^a^	1.000
Platelet < 100,000 count/mm^3^ (%)	12 (9.8) ^a^	1 (8.3)	11 (9.9) ^a^	1.000
CRP ≥ 40 g/L (%)	35 (30.7) ^b^	8 (66.7)	27 (26.5) ^b^	0.012*
PCT (μg/L), median(IQR)	0.37 (0.14–2.08) ^c^	19.11 (1.97–42.31) ^d^	0.3 (0.13–1.56)^e^	< 0.001*
GPT > 40 U/L (%)	44 (47.3) ^f^	6 (60) ^g^	38 (45.8) ^h^	0.509
GOT > 40 U/L (%)	62 (66.7) ^f^	5 (50) ^g^	57 (68.7)^h^	0.292

*CRP*, C-reactive protein; *PCT*, procalcitonin; *GPT*, glutamic-pyruvic transaminase; *GOT*, glutamic-oxaloacetic transaminase

^a^7 cases were missing; ^b^16 cases were missing; ^c^31 cases were missing; ^d^4 cases were missing; ^e^27 cases were missing; ^f^37 cases were missing; ^g^2 cases were missing; ^h^35 cases were missing

^*^P < 0.05

**Table 2:** Risk factors for children with meningitis caused by *Salmonella* infection. Univariate analysis

Variables	Meningitis group	Non-meningitis group	OR (95%CI)	*P* value
	(*n* = 12)	(*n* = 118)		
Age < 12 months	11 (91.7)	48 (40.7)	16.04 (2–128.38)	0.009*
Septic shock	2 (16.7)	1 (0.8)	23.4 (1.95–281.05)	0.013*
Vomit	5 (41.7)	6 (5.1)	13.33 (3.25–54.68)	< 0.001*
Convulsion	6 (50)	7 (5.9)	15.86 (4.05–62.11)	< 0.001*
CRP ≥ 40 g/L	8 (66.7)	27 (26.5)^a^	5.56 (1.55–19.95)	0.009*
PCT (μg/L)	19.11 (1.97–42.31) ^b^	0.3 (0.13–1.56)^c^	1.05 (1.01–1.1)	0.013*

^a^16 cases were missing; ^b^4 cases were missing; ^c^27 cases were missing

^*^P < 0.05

The median length of hospital stay was 10 (IQR: 7–15.25, range: 1–80) days. Nine (6.9%) children were admitted to pediatric intensive care. One hundred and thirteen (86.9%) cases recovered or improved, 5 (3.8%) cases were deceased or hopeless discharged, and 12 (9.2%) cases were transferred to a different hospital or left against medical advice. The patients’ accompanying comorbidities included pneumonia (33/130, 25.4%), liver disease (9/130, 6.9%), cardiac disease (4/130, 3.1%), hematological malignancies (7/130, 5.4%), prematurity (3/130, 2.3%), congenital malformations (2/130, 1.5%), nephrotic syndrome (1/130, 0.8%), traumatic injury (1/130, 0.8%), and acute suppurative appendicitis with perforation (1/130, 0.8%). Thirty-nine (30%) patients had more than one comorbidity.

The initial empirical antibiotic therapy included 3rd-generation cephalosporins or in combination with β-lactam (ceftriaxone, ceftazidime, ceftizoxime, cefoperazone-sulbactam) in 70 (53.8%) patients, penicillins or in combination with β-lactam (mezlocillin, mezlocillin-sulbactam) in 31 (23.8%) patients, 1st- or 2nd-generation cephalosporins in 11 (8.5%) patients, carbapenems in 10 (7.7%) patients, macrolides in 7 (5.4%) patients, and aztreonam in 1 (0.8%) patient. Some patients were given more than one antibiotic (3rd-generation cephalosporin-based, in combination with macrolides or vancomycin or linezolid). Of those receiving initial penicillins or in combination with β-lactam, and 1st- or 2nd-generation cephalosporins, 17 were switched to 3rd-generation cephalosporins or carbapenems.

### Serovar distribution of invasive *Salmonella* infections

The distribution of all the invasive *Salmonella* serovars is displayed in Table 3. All of the 5 *Salmonella* Paratyphi isolates were from Hainan Women and Children’s Medical Center in the south and 54.5% (6/11) of *Salmonella* Typhi isolates were from the First Affiliated Hospital of Guangxi Medical University in the south. The clinical characteristics of invasive *Salmonella* infections stratified by serovars are shown in Table 4. The patients with invasive NTS infections were younger than those with invasive *Salmonella* Typhi and *Salmonella* Paratyphi infections (*P* = 0.001). Moreover, the presence of comorbidities was significantly higher among those with invasive *Salmonella* Typhi and *Salmonella* Paratyphi infections (*P* = 0.005). There was no statistical difference in clinical symptoms on admission between the two groups. Patients with *Salmonella* Typhi and *Salmonella* Paratyphi infections had the higher frequencies of CRP ≥ 40 g/L (*P* = 0.003) and platelet < 100,000 count/mm^3^ (*P* = 0.040) as well as the higher PCT (*P* = 0.013), compared to the NTS group.

**Table 3:** The surveillance cohort of invasive *Salmonella* infections stratified by *Salmonella enterica* serovars

	No. of cases	Jiangxi Provincial Children’s Hospital, no	Henan Children’s Hospital, no	West China Women’s and Children’s Hospital, no	Xiamen Children’s Hospital, no	Hainan Women and Children’s Medical Center, no	The First Affiliated Hospital of Guangxi Medical University, no	Guangzhou Women and Children’s Medical Center, no	Hangzhou Children’s Hospital, no	Qingdao Women and Children’s Hospital, no	The Forth Hospital of Baotou, no
*S.* Typhi	11	2	0	1	0	0	6	1	1	0	0
*S.* Paratyphi	5	0	0	0	0	5	0	0	0	0	0
NTS											
Group B	10	0	0	3	3	0	0	1	2	1	0
Derby	2	0	0	1	0	0	0	0	1	0	0
Typhimurium	3	0	0	1	1	0	0	1	0	0	0
Group C	8	2	3	1	2	0	0	0	0	0	0
Choleraesuis	5	2	3	0	0	0	0	0	0	0	0
Group D	11	0	0	6	0	1	0	3	0	1	0
Enteritidis	7	0	0	3	0	1	0	2	0	1	0
Dublin	4	0	0	3	0	0	0	1	0	0	0
Group E	3	0	0	0	3	0	0	0	0	0	0
Meleagridis	1	0	0	0	1	0	0	0	0	0	0
Other serovars	82	40	32	3	1	3	1	0	1	0	1

*S. Typhi*, *Salmonella* Typhi; *S. Paratyphi*, *Salmonella* Paratyphi; *NTS*, nontyphoidal *Salmonella*

**Table 4:** The clinical characteristics of invasive *Salmonella* infections stratified by *Salmonella* serovars

Parameters	Total	*Salmonella* Typhi and Paratyphi	Nontyphoidal *Salmonella*	*P* value
	(*n* = 130)	(*n* = 16)	(*n* = 114)	
*Demographics*				
Age (month), median (IQR)	12 (8–20.25)	30 (14.3–48.5)	11.5 (8–16.5)	0.001*
Gender, male (%)	77 (59.2)	11 (68.8)	66 (57.9)	0.408
Comorbidities (%)	55 (42.3)	12 (75)	43 (37.7)	0.005*
*Clinical diagnosis*				
Meningitis (%)	12 (9.2)	1 (6.3)	11 (9.6)	1.000
Septic arthritis (%)	5 (3.8)	0 (0)	5 (4.4)	1.000
*Symptoms on admission*				
Fever (%)	118 (90.8)	15 (93.8)	103 (90.4)	1.000
Fever duration (day), median (IQR)	5 (2–7)	6 (3–11)	4 (2–7)	0.085
Septic shock (%)	3 (2.3)	0 (0)	3 (2.6)	1.000
Rash (%)	15 (11.5)	2 (12.5)	13 (11.4)	1.000
Cough (%)	37 (28.5)	8 (50)	29 (25.4)	0.072
Respiratory distress (%)	5 (3.8)	0 (0)	5 (4.4)	1.000
Tachypnea (%)	8 (6.2)	1 (6.3)	7 (6.1)	1.000
Cyanosis (%)	2 (1.5)	0 (0)	2 (1.8)	1.000
Vomit (%)	11 (8.5)	2 (12.5)	9 (7.9)	0.625
Diarrhea (%)	43 (33.1)	2 (12.5)	41 (36)	0.062
Convulsion (%)	13 (10)	3 (18.8)	10 (8.8)	0.201
*Laboratory indicators on admission*				
Leukocyte < 4000 or > 15,000 count/mm^3^ (%)	43 (35) ^a^	8 (53.3)^b^	35 (32.4)^c^	0.111
Neutropenia (%)	8 (6.5) ^a^	3 (20)^b^	5 (4.6) ^c^	0.057
Platelet < 100,000 count/mm^3^ (%)	12 (9.8) ^a^	4 (26.7)^b^	8 (7.4) ^c^	0.040*
CRP ≥ 40 g/L (%)	35 (30.7) ^d^	10 (66.7)^b^	25 (25.3)^e^	0.003*
PCT (μg/L), median(IQR)	0.37 (0.14–2.08) ^f^	1 (0.56–5.62) ^g^	0.29 (0.13–1.8) ^h^	0.013*
GPT > 40 U/L (%)	44 (47.3) ^i^	5 (33.3) ^b^	39 (50) ^j^	0.236
GOT > 40 U/L (%)	62 (66.7) ^i^	9 (60) ^b^	53 (67.9) ^j^	0.550

^a^7 cases were missing; ^b^1 case was missing; ^c^6 cases were missing; ^d^16 cases were missing; ^e^15 cases were missing; ^f^31 cases were missing; ^g^3 cases were missing; ^h^28 cases were missing; ^i^37 cases were missing; ^j^36 cases were missing

^*^P < 0.05

All of the 11 *Salmonella* Typhi isolates were recovered from the blood. One *Salmonella* Paratyphi isolate was recovered from cerebrospinal fluid and the other 4 *Salmonella* Paratyphi isolates were recovered from the blood. Eight isolates from cerebrospinal fluid were NTS including one *Salmonella enteritidis* serovar, one group E serovar, and six other serovars. The isolate from joint fluid was *Salmonella enteritidis* and the isolate from bone aspirate was other NTS serovars (Table 5).

**Table 5:** The distribution of *Salmonella enterica* serovars stratified by the sites of invasive *Salmonella* infections

	Blood, *n* (% of *N*)	Cerebrospinal fluid, *n* (% of *N*)	Joint fluid, *n* (% of *N*)	Bone aspirate, *n* (% of *N*)
Total number, *N*	119 (100)	9 (100)	1 (100)	1 (100)
*S.* Typhi	11 (9.2)	0 (0)	0 (0)	0 (0)
*S.* Paratyphi	4 (3.4)	1 (11.1)	0 (0)	0 (0)
NTS	104 (87.4)	8 (88.9)	1 (100)	1 (100)
Group B	10 (8.4)	0 (0)	0 (0)	0 (0)
Derby	2 (1.7)	0 (0)	0 (0)	0 (0)
Typhimurium	3 (2.5)	0 (0)	0 (0)	0 (0)
Group C	8 (6.7)	0 (0)	0 (0)	0 (0)
Choleraesuis	5 (4.2)	0 (0)	0 (0)	0 (0)
Group D	9 (7.6)	1 (11.1)	1 (100)	0 (0)
Enteritidis	5 (4.2)	1 (11.1)	1 (100)	0 (0)
Dublin	4 (3.4)	0 (0)	0 (0)	0 (0)
Group E	2 (1.7)	1 (11.1)	0 (0)	0 (0)
Meleagridis	1 (0.8)	0 (0)	0 (0)	0 (0)
Other serovars	75 (63)	6 (66.7)	0 (0)	1 (100)

### Antimicrobial resistance of invasive *Salmonella* isolates

A total of 127 *Salmonella* isolates were tested for antimicrobial susceptibility. The resistance patterns of invasive *Salmonella* isolates to antimicrobial agents are shown in Table 6. There were 50% of the *Salmonella* Typhi isolates resistant to ampicillin or piperacillin, followed by ampicillin/sulbactam (20%), chloramphenicol (20%), sulfamethoxazole/trimethoprim (9.1%), and cefepime (9.1%). However, the NTS serovars had high rates of resistance to ampicillin (57.3%), ampicillin/sulbactam (54.7%), piperacillin (45.3%), and tetracycline (32%). All of the *Salmonella* Typhi, *Salmonella* Paratyphi, and NTS serovars were susceptible to tested carbapenems (such as imipenem, meropenem, or ertapenem). Furthermore, we detected that the *Salmonella* Paratyphi isolates exhibited high rates of resistance to ciprofloxacin (66.7%) and ampicillin (40%). In total, 26 (20.5%) isolates were MDR. There was no MDR detected in the *Salmonella* Typhi group. The MDR rates of *Salmonella* Paratyphi and NTS serovars were 20% and 22.5%, respectively (see Table 6).

**Table 6:** Resistance patterns of invasive *Salmonella* isolates to antimicrobial agents

Antimicrobial drugs	Percentage resistance % (number of isolates resistant/number of isolates tested)		
	*Salmonella* Typhi	*Salmonella* Paratyphi	Nontyphoidal *Salmonella*
Ampicillin	50 (5/10)	40 (2/5)	57.3 (63/110)
Piperacillin	50 (4/8)	NT	45.3 (29/64)
Ampicillin/sulbactam	20 (1/5)	NT	54.7 (58/106)
Piperacillin/tazobactam	0 (0/11)	NT	2.8 (3/106)
Cefotaxime or ceftriaxone	0 (0/11)	20 (1/5)	9.9 (11/111)
Ceftazidime	0 (0/10)	20 (1/5)	7.2 (8/111)
Cefepime	9.1 (1/11)	0 (0/5)	5.5 (6/110)
Aztreonam	0 (0/11)	NT	7.3 (8/109)
Gentamicin or tobramycin	0 (0/5)	NT	18.5 (20/108)
Amikacin	0 (0/5)	NT	10.5 (11/105)
Imipenem	0 (0/11)	NT	0 (0/108)
Meropenem	0 (0/8)	NT	0 (0/88)
Ertapenem	0 (0/2)	0 (0/5)	0 (0/32)
Tetracycline	0 (0/2)	NT	32 (24/75)
Ciprofloxacin or levofloxacin	0 (0/9)	66.7 (2/3)	11.2 (11/98)
Sulfamethoxazole/trimethoprim	9.1 (1/11)	20 (1/5)	24.8 (27/109)
Chloramphenicol	20 (1/5)	NT	16 (12/75)
MDR	0 (0/11)	20 (1/5)	22.5 (25/111)

*NT*, not test; *MDR*, multidrug resistance

## Discussion

This study is meaningful not only for its clinical purpose but also for its regional distribution in China. The clinical data from the 10 hospitals located in different regions during a certain period from 2016 to 2018 reliably represent the major demographic and clinical characteristics of children who suffered from invasive salmonellosis in China. Obviously, NTS other than *Salmonella* Typhi or *Salmonella* Paratyphi has been the major cause of invasive *Salmonella* infections in Chinese children. The national prevalence of typhoid and paratyphoid in China declined with year since 2008[9]. We also found that invasive typhoid and paratyphoid in this case cohort were mostly from Hainan province and Guangxi province, which is characteristic of warm and humid tropical climate with economy and culture relatively underdeveloped. Besides, the prevalence of antimicrobial resistance among NTS was higher than among *Salmonella* Typhi or Paratyphi. The data of antimicrobial resistance will be useful to help guide the appropriate use of empirical antibiotics and improve the clinical outcome.

The majority of NTS infections occurred between May and October. The seasonal trend is consistent to that of NTS gastroenteritis reported by national laboratory surveillance network [10, 16]. Seasonal variation was reported in other countries. In Mali, invasive NTS infection appeared most frequently from August to November and peaked in October, toward the middle and end of the rainy season [13]. In Malaysian Borneo, no significant correlation was observed in invasive *Salmonella* infection among children between the monthly incidence and average monthly rainfall [17].

We observed that 85.4% of cases occurred in children younger than 36 months old with the median age of 12 months. Meanwhile, we also noticed that children suffering from invasive NTS disease are younger than those suffering from non-invasive NTS gastroenteritis in Chinese children (the median age: 18 months) [18]. Generally, the age distribution of case series in Chinese children is similar to that reported in other developed and developing countries. In Africa, infants aged 6–11 months and toddlers aged 12–23 months exhibit the highest incidence of severe invasive NTS disease [19]. An Australian study also showed that age-related peaks in NTS infections were seen in infants, which was likely to be due to highly susceptible intestinal microflora in the setting of an immature immune system [20]. Thus, anti-infective therapy should be empirically initiated for young children who are at high risk of developing invasive salmonellosis.

Only 33% of cases presented with gastroenteritis, and even 9.2% of cases were afebrile. Of note, pneumonia was the common accompanying comorbidity in children with invasive salmonellosis. Most of African children with invasive NTS disease were reported to present with respiratory symptoms [21, 22]. In Ghana, NTS was shown to be the predominant organism isolated in children with clinical pneumonia [23]. In Malaysian Borneo, pneumonia was the primary presentation, seen in 76% of children with *Salmonella enteritis* bacteremia [17]. In Thailand, clinical pneumonia was diagnosed in 25% of children with invasive NTS disease [24]. Taken together, the manifestation of invasive salmonella infection was nonspecific. Besides, we noticed 9.2% of cases were complicated with serious meningitis and arthritis. Infants were much more likely to develop meningitis compared to children ≥ 12 months of age. In addition to young age, the presentations of septic shock, vomit, convulsion, CRP ≥ 40 g/L, and higher level of PCT on admission were statistically related to an increased risk of developing meningitis. These warning parameters will help clinical decision on how to proceed the management and investigation of suspected *Salmonella* infection in a timely manner. In regions with a high prevalence of invasive NTS infection, *Salmonella* has been reported to be the leading cause of septic arthritis in children [25]. In this current study, septic arthritis secondary to NTS infection was not common and seen in 3.8% of patients. However, NTS should not be neglected as a pathogen of septic arthritis among young children*.*

In this survey, the patients in the NTS group were younger than those in the *Salmonella* Typhi and *Salmonella* Paratyphi group (*P* = 0.001). The presence of comorbidities was clearly higher among those infected by *Salmonella* Typhi and *Salmonella* Paratyphi (*P* = 0.005). But there was not any statistical significance between the two groups regarding gender or symptoms on admission such as fever, septic shock, rash, cough, respiratory distress, tachypnea, cyanosis, vomit, diarrhea, and convulsion. Although the clinical manifestations appeared to be nonspecific, some meaningful laboratory factors were discovered in the *Salmonella* Typhi and *Salmonella* Paratyphi group such as the presence of CRP ≥ 40 g/L (*P* = 0.003) and platelet < 100,000 count/mm^3^ (*P* = 0.04) as well as higher PCT (*P* = 0.013) compared to the NTS group. Because of the insufficient samples in the fatal group, we could not step to perform the multivariable analysis and this remains a limitation of our study. Although the reported case-fatality rate of invasive NTS infections in Mali and Vietnam was as high as 20.3 to 26% [8, 19], the clinical outcome is better in Chinese children with about 4% of case-fatality rate. The better outcome in Chinese children is attributable to timely empirical antibiotic initiation in the hospital, as well as less immunocompromised children in this case series.

When choosing empiric treatment, the local prevalence of antimicrobial resistance should be considered. Currently, MDR *Salmonella* Typhi is considered endemic in many developing countries, especially in areas of South and Southeast Asia, even in the Netherlands [26]. However, we found no MDR in the *Salmonella* Typhi group. Chloramphenicol, ampicillin, and sulfamethoxazole-trimoxazole had ever been the first-line antibiotics for the treatment of salmonellosis since the 1970s, but fell in disuse because of resistance or toxicity concerns. So far, ampicillin resistance remains at the high level in many developing countries [19, 27, 28], but at the low level in some developed countries, for example, in Australia and England, where the prevalence of ampicillin was reported at 9.9% and 17.3% [16, 20]. In this current study, the prevalence of ampicillin and ampicillin/sulbactam resistance among *Salmonella* isolates remained high, similar to the previous studies from China [18, 29, 30]. For children, the third-generation cephalosporins are the preferred choice for the treatment of typhoid fever and severe NTS infections [31]. However, we noticed that 20% of *Salmonella* Paratyphi isolates and 9.9% of the NTS isolates displayed resistance to cefotaxime or ceftriaxone, which is similar to the situation reported in Kenya [32]. In other countries such as England, Vietnam, and the USA, the *Salmonella* resistance is at the low level with the reported prevalence of 0.9 to 3% [16, 27, 33]. Ciprofloxacin is also recommended as the first-line regimen for the treatment of enteric fever and severe NTS infection by the WHO [31]. However, there is an increasing trend of fluoroquinolone resistance in many areas, mostly in Asia [34]. In the current study, the resistances to ciprofloxacin in the *Salmonella* Typhi, *Salmonella* Paratyphi, and NTS isolates were 0, 66.7%, and 11.2%, respectively. In a study conducted in Shanghai, 61.8% of NTS isolates showed decreased ciprofloxacin susceptibility [12]. Similarly, increasing ciprofloxacin resistance was observed from 2012 to 2017 (4.2 to 22.0%) in Zimbabwe [35]. In European countries, the proportion of *Salmonella* isolates resistant to ciprofloxacin significantly increased to 12.5% in 2018 [36]. NTS resistance rate in NTS blood isolates in the UK was up to 20.7% [16]. The percentage of *Salmonella* Typhi non-susceptible to ciprofloxacin reached 74% but NTS resistance percentage in NTS was only 7% in 2017 in the USA [33]. These data might be helpful for us to choose empirical antibiotics according to different serovars and patient location in clinical practice. Salmonellosis is now a common disease for travelers.

Although this multicenter study reported a pooled case series, it still has a few limitations. Firstly, some NTS isolates were unclassified as well as some clinical data was missing. Secondly, we notice the potential bias of regional case distribution owing to some clinical factors, especially overuse broad-spectrum antibiotics for febrile children prior to hospitalization with no blood culture testing. Even so, we still feel that inclusion of a few cases from these hospitals can at least reflect the current real clinical picture of invasive *Salmonella* infections in China. Thirdly, due to the retrospective study limitation, we could not obtain the isolates from the different hospitals to perform laboratory-based measurement. However, we adjusted the MIC data of isolates according to the Clinical and Laboratory Standards Institute (CLSI) performance guideline and re-affirmed the original results of antimicrobial susceptibility testing. Fourthly, antimicrobial susceptibility to azithromycin was not evaluated in all participating hospitals, which is recommended as the first choice for the treatment of enteric fever and invasive NTS-associated diarrhea [31]. Notwithstanding these limitations in this retrospective study, our findings provide a comprehensive description to date of clinical characteristics and antimicrobial susceptibility of pediatric invasive *Salmonella* infection from ten provinces across China. For invasive salmonellosis, as an important clinical and public health issue, continued surveillance should be performed.
